# Insights into evolving global populations of *Phytophthora infestans* via new complementary mtDNA haplotype markers and nuclear SSRs

**DOI:** 10.1371/journal.pone.0208606

**Published:** 2019-01-02

**Authors:** Frank N. Martin, Yonghong Zhang, David E. L. Cooke, Mike D. Coffey, Niklaus J. Grünwald, William E. Fry

**Affiliations:** 1 USDA-ARS, Crop Improvement and Protection Research Unit, Salinas, California, United States of America; 2 Plant Pathology and Microbiology Department, University of California, Riverside, California, United States of America; 3 The James Hutton Institute, Invergowrie, Dundee, Scotland; 4 USDA-ARS, Horticultural Crops Research Laboratory, Corvallis, Oregon, United States of America; 5 Plant Pathology and Plant-Microbe Biology Section, School of Integrative Plant Science, Cornell University, Ithaca, New York, United States of America; Agriculture and Agri-Food Canada, CANADA

## Abstract

In many parts of the world the damaging potato late blight pathogen, *Phytophthora infestans*, is spread as a succession of clonal lineages. The discrimination of genetic diversity within such evolving populations provides insights into the processes generating novel lineages and the pathways and drivers of pathogen evolution and dissemination at local and global scales. This knowledge, in turn, helps optimise management practices. Here we combine two key methods for dissecting mitochondrial and nuclear diversity and resolve intra and inter-lineage diversity of over 100 *P*. *infestans* isolates representative of key clonal lineages found globally. A novel set of PCR primers that amplify five target regions are provided for mitochondrial DNA sequence analysis. These five loci increased the number of mtDNA haplotypes resolved from four with the PCR RFLP method to 37 (17, 6, 8 and 4 for Ia, Ib, IIa, and IIb haplotypes, respectively, plus 2 Herb-1 haplotypes). As with the PCR RFLP method, two main lineages, I and II were defined. Group I contained 25 mtDNA haplotypes that grouped broadly according to the Ia and Ib types and resolved several sub-clades amongst the global sample. Group II comprised two distinct clusters with four haplotypes corresponding to the RFLP type IIb and eight haplotypes resolved within type IIa. The 12-plex SSR assay revealed 90 multilocus genotypes providing accurate discrimination of dominant clonal lineages and other genetically diverse isolates. Some association of genetic diversity and geographic region of contemporary isolates was observed; US and Mexican isolates formed a loose grouping, distinct from isolates from Europe, South America and other regions. Diversity within clonal lineages was observed that varied according to the age of the clone. In combination, these fine-scale nuclear and maternally inherited mitochondrial markers enabled a greater level of discrimination among isolates than previously available and provided complementary perspectives on evolutionary questions relating to the diversity, phylogeography and the origins and spread of clonal lineages of *P*. *infestans*.

## Introduction

*Phytophthora infestans* is the cause of potato late blight and other devastating diseases of solanaceous crops. The pathogen has spread across the globe from its center of origin in central [1], [2] or South America, to North America, Europe and beyond [3], [4], [5, 6]. This algal-like oomycete is a diploid heterothallic species, requiring the association of A1 and A2 mating types to form long-lived sexual oospores. In some regions such as Mexico [7], [2], [8] and subsequently in parts of Europe [9] sexual progeny from germinated oospores are an important source of primary inoculum generating genetically diverse populations. A series of epidemiologically successful clonal lineages that were distributed primarily by trade in solanaceous plants (seed tubers or young plants) have dominated in many key production areas [10]. Some of these clonal lineages have been shown to be triploid [11]. The FAM-1 lineage with a HERB-1 mtDNA haplotype was recently proposed as the primary wave of infection to North America and Europe in the nineteenth century [11], [4], [5, 12] that culminated in potato crop failure and the great famines in Ireland and other parts of Europe [13],[14]. FAM-1 was displaced by the US-1 lineage which dominated global populations for decades and remains prevalent in some regions [11], [15], [16]. The major US-1 migration was presumed to consist of only the A1 mating type, thus precluding sexual reproduction, but subsequent migrations into Europe and USA from Mexico included both mating types and genetic diversity has increased. Within such mixed populations, however clonal lineages such as US-8 and, more recently, US-22 and US-23 in North America [17] and 13_A2 in Europe [18], [19] have emerged to become locally dominant [10]. Tracking the evolutionary drivers and change in pathogen populations has practical short and longer-term benefits; it informs local disease management practices and ensures effective longer-term strategic planning to curb the emergence of populations that overcome host resistance or are less sensitive to fungicide active ingredients.

The diversity of *P*. *infestans* populations has been studied with a wide variety of phenotypic and genotypic markers reviewed by Cooke and Lees [20]. Lineages have been defined by a combination of mating type, isozymes, mtDNA haplotyping and RFLP analysis with the RG57 probe [21]. More recently, simple sequence repeats (SSRs) have become the markers of choice for analysis of population structure and genetic diversity [22]. Several sets of codominant markers are available for *P*. *infestans*, [23], [24], [25] the most appropriate of which have been combined into an efficient 12-plex SSR set [26]. Such SSRs have been used to discriminate and track contemporary and historic clonal lineages in the UK [18], the Netherlands [19], the US [17] and other regions [27], [28], [29], [11]. These SSR markers also reveal high levels of diversity in sexually recombining populations of *P*. *infestans* in the Nordic regions [30], [31] and Mexico [8]. They form a key element of the EuroBlight monitoring (www.euroblight.net) and USAblight (usablight.org). The SSRs can be used to query the *Phytophthora*-ID database [32] for rapid placement of any genotype based on multiplex SSR protocols using a reference database and reference strains specifically tailored to identifying US clonal lineages [33].

There are many advantages of using mtDNA to trace the evolutionary history or phylogeography of organisms; the mitochondrial genome acts as a uniparentally inherited non-recombining clone [34], [35], it is presumed to evolve in a nearly neutral manner and its relatively high and uniform mutation rate allow analysis of approximate divergence times [4], [5]. The first use of mitochondrial haplotypes for characterization of isolates of *P*. *infestans* was reported by Carter et al. [36] using RFLP analysis of purified mitochondrial DNA to identify 4 types of banding patterns. Two main groups were observed, with one group having a fragment 2 kb larger than the other (type II and type I, respectively). These groups were subsequently divided into subgroups "a" and "b" based on addition/loss of restriction sites. Griffith and Shaw [37] simplified this analysis by developing a PCR -RFLP based technique for the specific regions of the mitochondrial genomes associated with haplotype classification (labelled as P1 through P4). While these remain the primary criteria for haplotype classification, additional analysis using a combination of Southern analysis, PCR-RFLP or DNA sequence analysis has been done [7], [38], [39], [40], [41], [42]. The identification of additional polymorphisms among mitochondrial haplotypes from these studies indicates that the current haplotype classification scheme should be further expanded to improve the number of markers available for population studies.

The availability of the mitochondrial genome sequences for representatives of the four primary haplotypes (Ia, Ib, IIa and IIb) of *P*. *infestans* [43], [44] has allowed a comparative genomics approach to be used to further explore sequence polymorphisms in other regions of the mitogenome. The availability of data identifying regions of the mitochondrial genome of other *Phytophthora* spp. exhibiting more intraspecific polymorphisms (F. N. Martin, unpublished) provides additional loci to examine for sequence polymorphisms to further delineate haplotype classification. Mitochondrial DNA analysis has been a valuable tool to help reconstruct the ancestry of hybrid lineages such as *P*. *andina* [45], [46]. More recent analysis of whole mitogenomes has confirmed the deep split into the type I and II lineages and highlighted a greater diversity within the type I lineage [6]. Comparing accumulated mutations in dated herbarium and living samples has been used to establish an approximate timescale for evolutionary events in the divergence of genomes [4], [5, 6, 12] and provides a high resolution analysis of mitochondrial haplotypes. Increased availability and reducing costs of genome sequencing provides opportunities for additional mitogenome assembly. While this provides the ultimate haplotype resolution, it remains relatively expensive for the analysis of large numbers of individuals required for a population genomics approach.

Individually, SSRs and mitochondrial haplotypes are valuable tools for examining pathogen dispersal and the evolutionary history of *P*. *infestans* populations. In combination, however, they provide even greater power as complementary tools to determine the phylogeography, transmission and putative parentage of key destructive lineages of *P*. *infestans* in global populations. The overall aim of this study was to assess the mtDNA diversity and corresponding nuclear SSR profiles of a broad collection of *P*. *infestans* isolates sampled from geographically diverse locations. Specific objectives were to a) to re-examine mtDNA sequence diversity in relation to the previous identification of 4 haplotypes defined by PCR-RFLPs and to present additional mtDNA target regions and protocols, b) to demonstrate the discriminatory power of SSRs amongst global lineages of the pathogen and c) to examine the way these nuclear SSR markers and the maternally inherited mitochondrial haplotypes complement each other for population studies. This work was also presented in the context of recent analysis of the historical HERB-1 mtDNA lineage of *P*. *infestans*.

## Materials and methods

### Cultures

The isolates used in this study (S1 Table) were obtained from the World Oomycete Genetic Resource Collection at the University of California, Riverside (http://phytophthora.ucr.edu). They were specifically selected to maximise genetic diversity based on prior analysis (M.D. Coffey, unpublished) across a diverse sample of global populations to demonstrate the power of the complementary approach of mtDNA and SSR analysis.

Mating type was determined by pairing on rye agar medium with known mating types of *P*. *infestans* using standard techniques [47]. Cultures were grown in liquid cultures and DNA extracted as previously described [48]. Genotyping of isolates using RG-57 was completed as described in Goodwin et al. [7]. Classification of traditional mitochondrial haplotypes Ia, Ib, IIa and IIb was done using a PCR-RFLP technique with loci P1 through P4 according to Griffith and Shaw [37]. To provide a historical context, sequence data from isolates KM-177513 (recovered from Ireland, 1846) and M-0182898 (recovered from Germany prior to 1863) as reported in Yoshida et al. [4] were included in the analysis (isolate 06_3928A from England, 2006 was included as well; unfortunately sequence datasets were not complete for all loci for the remaining isolates from their study). Mitochondrial genome sequences from Martin et al. [5, 6, 11, 12] and Saville et al. [11] were not included in this analysis as they lacked data from locus 1 (*rpl5* to *rns*) of this current study. Two isolates of *P*. *mirabilis* were used as an outgroup (S1 Table).

### Mitochondrial loci for haplotyping

**Fig 1:**
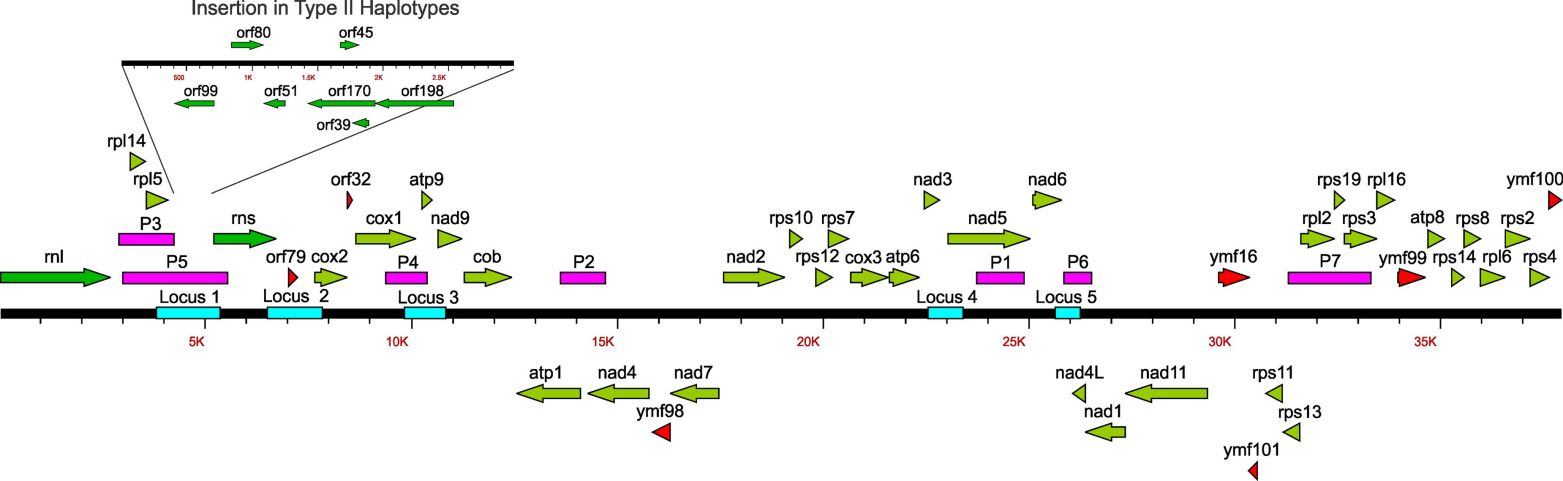
Linear map of the mitochondrial genome of *Phytophthora infestans* with the loci used for classification of mitochondrial haplotype. Regions P1 through P4 were reported by Griffith and Shaw [37] (red fill), P5 through P7 by Gavino and Fry [39] (red fill) and locus 1 through 5 (blue fill) used in this investigation.

A total of 5 loci were used for classification of mitochondrial haplotypes (Fig 1, shown in blue). These loci were selected based on comparative analysis of published mitochondrial genomes (NC002387 [43], AY894835, AY898627 and AY898628 [44]) and evaluation of regions of the mitochondrial genomes of other *Phytophthora* spp. exhibiting intraspecific polymorphisms with the final selection of loci to sequence based on the level of sequence divergence observed and lack of redundancy for haplotype classification (F. N. Martin, unpublished). Locus 1 spanned the intergenic region between *rpl5* and *rns*, which includes the 1.88 kb insertion associated with differentiating haplotype I and II (it is the distal portion of region P5 of Gavino and Fry [39]). In haplotype I examples the amplicon is approximately 1.3 kb while for haplotype II it is 3.2 kb. Locus 2 is approximately 1.1 kb and spans the 3' end of the *rns* coding region to the 5' end of the *cox2* gene and includes *orf79*. Locus 3 is approximately 0.85 kb and spans the 3' end of the *cox1* to the 5' end of *nad9* and includes the *atp9* gene (this includes a portion of the P4 region [37]). Locus 4 is approximately 0.4 kb and includes the *nad3* gene and the 5' end of *nad5*. Locus 5 is approximately 0.5 kb and spans the 3' end of *nad6* to *nad4L* (this includes a portion of the P6 region; [39]).

The primers used for amplification and sequencing of mitochondrial loci are listed in Table 1. All amplifications were done with approximately 10 ng template DNA, 0.5 μM forward and reverse primers, 2 mM MgCl_2_, 100 μM dNTP, 1x amplification buffer and 1 unit of AmpliTaq (Applied Biosystems, Foster City, CA) in a volume of 25 μl. Templates were amplified in an ABI 9600 thermal cycler with the following cycling conditions: 1 interval of 95° C for 3 min; 35 cycles of 95° C for 1 min, 1 min annealing for the indicated temperature (Table 2) and extension at 72° C for 2 min (3 min for locus 1); 1 interval of 72° C for 5 min followed by a 10° C hold. After confirming template amplification by running samples on an agarose gel, sequencing templates were prepared by treatment with ExoSap-IT (USB, Cleveland, OH) in accordance with the manufacturer’s instructions and sent to the Penn State Genomics Core Facility of the Huck Institute for the Life Sciences (University Park, Pennsylvania) for Sanger sequencing with the amplification primers unless otherwise noted (Table 1). Each template was sequenced in both directions to generate a consensus sequence based on complementary strands in Sequencher 4.7 (Gene Codes, Ann Arbor, MI). All sequences have been deposited in GenBank (S2 Table).

**Table 1:** Primers used for DNA amplification and sequencing of mitochondrial loci.

Target	Primer	Primer sequence(5’-3’)	Amplicon size (bp)	Annealing Temperature (°C)
**Region 1** *rp15-rns*	PiR1-F	AAGA TATACCTCCAATAGATG 3,985–4,005^1^	Haplotype I—1.3 kb Haplotype II—3.2 kb	56
	PiR1-R	GATAATTCACGTGTTACTCAC 5,308–5,288		
	PiR1-Fn1^2^	GTT TTATTTAAACCTTCTTCAG		
	PiR1-Fn2^2^	CTCATA TTTTGAATAAAGTCTC		
	PiR1-Fn3^2^	TTATATAACCAAAAACTACT TC		
	PiR1-Rn1^2^	ACCAAGATCAAATATTAGATCA		
	PiR1-Rn2^2^	AAGTTAGGACATATTTACATGA		
	PiR1-Rn3^2^	AAATTATCAAATAGGTAAACGAA		
**Region 2** *rns- cox2*	PiR2-F	AATTAGTAACT TTGATGAAGT C 6,638–6,659	1.1 kb	60
	PiR2-R	AAACCTAATTGCCAAGGTTC 7,731–7,712		
**Region 3** *cox1-nad9*	PiR3-F	AAATGTTACCTTTTTTCCAATG 9,925–9,946	0.85 kb	56
	PiR3-R	ATAGGAATTAATTTATCTGAAG 10,770–10,749		
**Region 4** *nad3-nad5*	PiR4-F	CATGGGGATTTTGGACTATG 22,745–22,764	0.4 kb	56
	PiR4-R	AAATACCGAACCTTTACGAC 23,145–23,126		
**Region 5** *nad6-nad4L*	PiR5-F	CCTTAATAGGTGCAGTAACT 25,674–25,693	0.5 kb	62
	PiR5-R	GTAGCAGCAGCAGAATCTG 26,166–26,148		

^1^ Base position in *P*. *infestans* mitochondrial genome NC002387

^2^ Nested primers used for sequencing of region 1.

### Haplotype analysis

Sequences for each region were aligned by Clustal W in Accelrys Gene v2.5 with McClade v. 4.02 (Sinaur Associates, Sunderland, MA) used to fine tune the alignment. Polymorphisms in the alignments among the isolates for each individual region were checked back against the trace files to ensure accurate base calling and classification as a separate haplotype. Once the analysis of the individual loci was completed and numerical classification of individual haplotypes done, the results for all 5 loci were combined and a numeral designation for each final multilocus haplotype was given (S1 Table). To obtain the final haplotype classification the individual alignments were concatenated into a single alignment file and analyzed using DnaSP 5.10.01 [49].

A haplotype network visualizing the relationship among the various haplotypes was calculated in SplitsTree ver 4.1 [50] using the unmodified dataset with uncorrected P and a NeighborNet network calculation. To reduce the complexity of the network a neighbor-joining network was constructed and 1,000 replicates of bootstrap analysis was run to determine statistical support for the branching. Network 4.5.1.6 (Fluxus Technology Ltd., Clare, Suffolk, England) was also used to confirm the network topography and overall grouping of haplotypes using maximum parsimony. A median joining network calculation [51] was completed using the default 1:1 transition: transversions ratio and epsilon = 0. After the first median joining network calculation was run the dataset underwent maximum parsimony post processing to delete links not used by the shortest trees in the network [52] and the network was redrawn.

### Phylogenetic analysis

A partition homogeneity test of the concatenated unmodified dataset was run in PAUP v4.0b10 and phylogenetic relationships were evaluated among isolates by several methods. PAUP v. 4.0b10 was used to conduct maximum parsimony (MP) analysis with a heuristic tree search with MULPARS on, steepest decent option off, random addition of sequences (10 replicates) and TBR branch swapping. To determine support for the various clades of the trees, the analysis was bootstrapped with 100 replicates. In view of the large dataset the maximum rearrangements allowed per replicate was 10^8^ to prevent the program from running out of memory. Maximum likelihood (ML) and Bayesian analyses (BA) were done using TOPALi ver. 2.5 [53] with JModelTest ver. 0.1.1 [54] used to determine the appropriate nucleotide substitution model. Based on the values of the Akaike Information Criterion, Bayesian Information Criterion and Decision Theory Performance Based Selection for the concatenated dataset, the transition nucleotide substitution model was selected (TIM + G). ML analysis was run in TOPALi using PhyML ver 2.4.5 with 100 bootstrap replicates. BA was run in TOPALi using MrBayes ver 3.1.1; two analyses were run simultaneously for 1,000,000 generations with a 20% sampling frequency and burn in of 40%. The runs converged after 1,000,000 generations with the potential scale reduction factor having a value of 1.0.

### SSR characterization

For each sample 12 SSR loci were amplified according to a previously published multiplex protocol [26] that amplified all 12 markers simultaneously and run on a 3730 capillary DNA analyzer according to the manufacturers default settings (Life Technologies, Grand Island, NY). The allele peaks were scored in GeneMapper 3.7 (Life Technologies) and exported to a MS Excel spreadsheet.

### Data analysis

The 103 samples were assigned to four locations as follows; 42 isolates sampled from a geographically defined Europe that included Russia (EU), 42 isolates sampled from North America (NA), 10 sampled from South America (SA) and 9 sampled from other regions of the world or representing progeny from laboratory crosses (‘Other’). The presence of three alleles at one or more SSR loci in many isolates was taken as evidence of increased ploidy. Bruvo distances are appropriate for the genetic analysis of data in which individuals of differing ploidy levels are observed [55], [56] and this approach was previously applied to populations of *P*. *infestans* [8]. Bruvo distances were calculated in *Polysat* [57] and a neighbor-joining tree generated using PHYLIP neighbor version 3.696 [58] before visualization in FigTree 1.4.2 (http://tree.bio.ed.ac.uk/software/figtree/) and annotation in Adobe Illustrator. The *poppr* R package version 2.0.2 [56] was used to generate the bootstrap values placed on the PHYLIP tree, the minimum spanning network, the genotype accumulation curve and measures of marker and population diversity. Principal co-ordinate analysis was completed in *Polysat* [57] implemented in R with the PCA and eigenvalues generated using the *cmdscale* command, plotted using GenStat for Windows 16^th^ Edition (VSN International, 2013) and annotated in Adobe Illustrator.

## Results

### Mitochondrial haplotype classification

The sequence-based methods in this study identified 37 mitochondrial haplotypes amongst the multiple isolates of *P*. *infestans* and a distinct one for a single isolate of *P*. *andina* (S1 Table). The most polymorphic locus was the 1.1 kb *rns* to *cox2* region (locus 2) with 18 haplotypes. There were seven SNPs (five conserved across type I and II haplotypes), ten indels (four 1 bp, one 2 bp, one 3 bp, one 4 bp, two 6 bp and one 24 bp) and different numbers of a 36 bp repeat (one, two, three or four copies located between *orf79* and *cox2*). The next most polymorphic locus was *rpl5* through to *rns* (locus 1), the region containing the 2.2 kb insertion discriminating type I from II haplotypes, with 8 haplotypes. In addition to this indel there were 10 SNPs observed, seven of which differentiated type I and II haplotypes. Two of the haplotypes were represented by single isolates, one of which was the 1846 collected isolate from Ireland (KM177513) and the 2006 isolate from England (063928A); these varied from the rest of the haplotypes by a different single base indel.

The 0.5 kb region spanning the *nad6* to *nad4L* genes (locus 5) had seven haplotypes consisting of two, one base indels and four SNPs (one conserved between type I and II haplotypes) while the 0.85 kb region spanning the *cox1* to *nad9* genes (locus 3) had six different haplotypes due to six SNPs. The least polymorphic was the 0.4 kb *atp6* through *nad5* (locus 4) with 3 mitochondrial haplotypes for all contemporary isolates. An additional 2 haplotypes were observed in the historical isolates from Ireland (KM177513) and Germany (M-1082898). These differed from the other mitochondrial haplotypes by a 5 bp indel (KM177513) and a second copy of a 13 bp repeat (M0182898).

**Fig 2:**
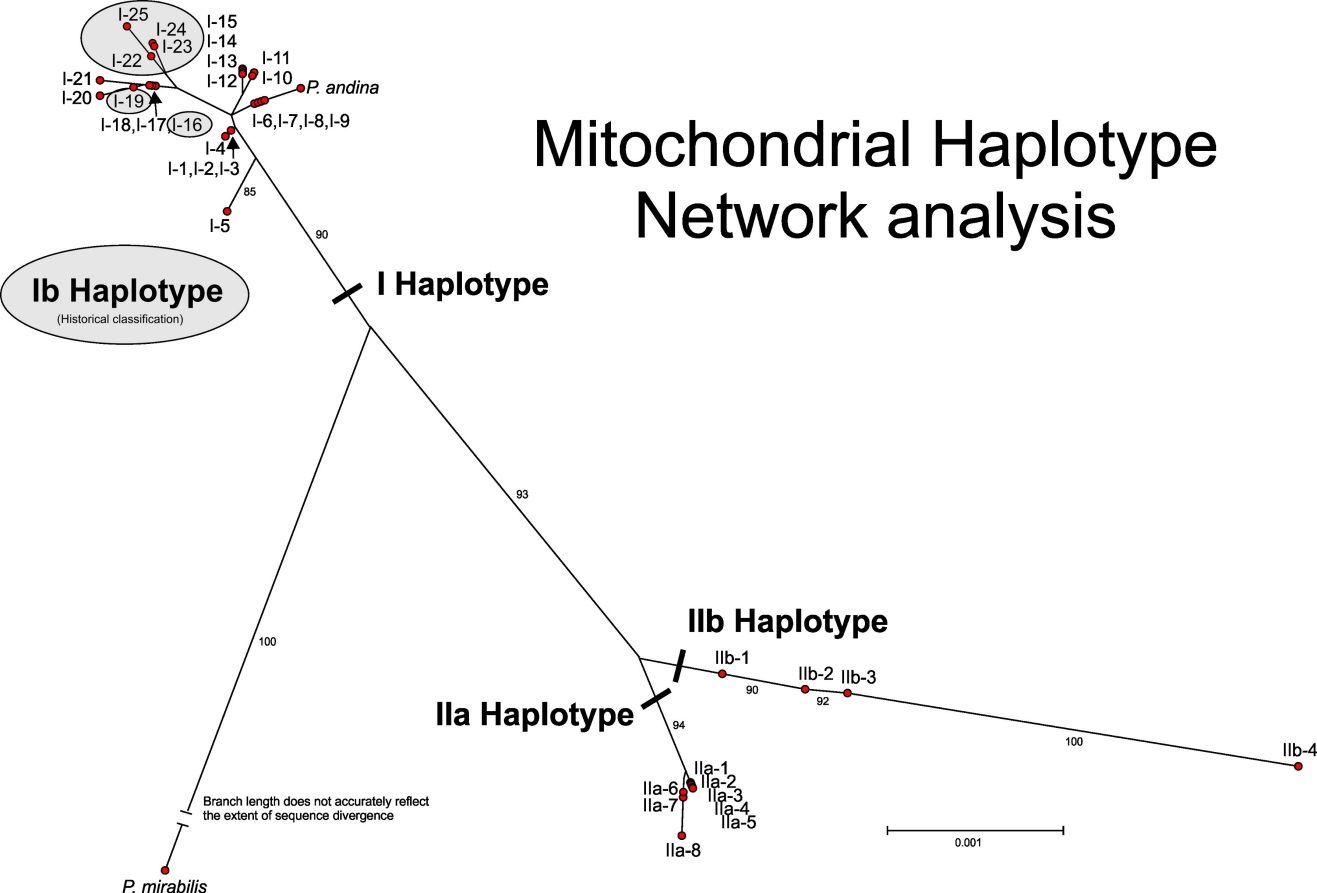
Mitochondrial haplotype network for *Phytophthora infestans* calculated by SplitsTree 4.10 [50] using uncorrected P with gaps included in the analysis and a neighbor joining network. Bootstrap support for main clades (1000 replicates) indicated by the smaller numbers on specific branches. Haplotypes in Haplogroup I not shaded represent Ia haplotypes.

Multilocus haplotype analysis of the concatenated data for all five loci (6,175 bp alignment) generated a total of 37 *P*. *infestans* multilocus mitochondrial haplotypes leading to a need for changing the historical haplotype naming convention. In this study we suggest adopting the terminology used for human mtDNA analysis whereby the major lineages are termed haplogroups and within each haplogroup the haplotypes are numbered [59]. Due to the clear separation of IIa and IIb haplogroups in the network analysis (Fig 2) with strong bootstrap support, this terminology was retained with the different haplotypes in each group denoted by an additional number (1 through 8 for IIa and 1 through 4 for IIb). Since there was no clear delineation of the haplotypes Ia and Ib defined by RFLPs all these isolates were grouped into a single haplogroup I with numbers ranging from 1 through 25. In addition to this labelling, a more detailed naming convention that reflects the haplotype classification at each individual locus is presented in S1 Table. This comprises the main haplogroup label followed sequentially by the haplotype classification for each individual locus. For example, haplogroup I-1 would be I-4-8-4-1-5 with locus 1 classified as haplotype 4, locus 2 classified as haplotype 8, etc.

While most isolates identified by the RFLP assay as haplotype Ib grouped within a cluster of haplotypes (I-22 to I-25), two (I-16 and I-19) were grouped among isolates typed by PCR-RFLP as haplotype Ia (Fig 2). Haplogroup I was most prevalent with 25 haplotypes amongst 59 isolates compared to IIa with eight haplotypes among 31 isolates and IIb with four haplotypes among eight isolates. Within haplogroup I a single group dominated with 18 of the 59 isolates (30%) having the closely related types (I-12 to I-15). In the network analysis (Fig 2) these haplotypes were tightly clustered together with the primary difference among them being the number of 36 bp repeats observed in the spacer between *rns* and *cox2* (locus 2). This variation in the number of repeats was also the primary difference for other tightly clustered haplotypes (I-6 through -9, I-10 and -11, I-12 through 15, and IIa-6 and -7).

### Phylogenetic analysis

**Fig 3:**
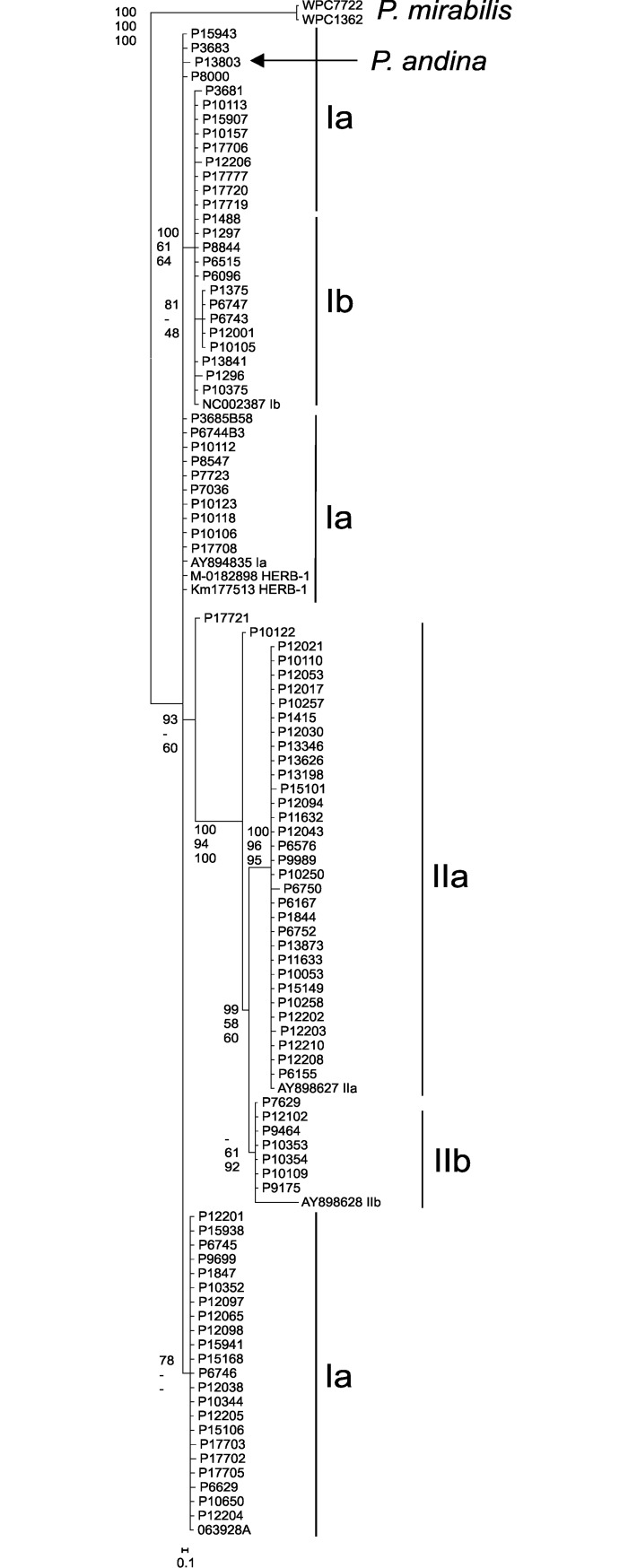
**Maximum parsimony tree of 6.175 kb concatenated alignment of the mitochondrial sequences from five loci used in classification of haplotypes with the bootstrap values at the nodes reflective of values obtained in maximum parsimony (top), maximum likelihood (middle) and Bayesian analysis (posterior possibilities; bottom).** Partition homogeneity test results for the combined dataset was 0.06.

In both the phylogenetic analysis (length mutations not considered in the analysis; Fig 3) and network analysis (length mutations are considered in the analysis; Fig 2) using the mitochondrial haplotype sequence data there is strong bootstrap support separating the outgroup *P*. *mirabilis* from *P*. *infestans*. *P*. *andina* isolate P13803 grouped with haplotype I-6, -7, -8 and -9 in the network analysis; haplotype I-6 and I-7 are representatives of the HERB-1 genotype. With *P*. *mirabilis* as an outgroup there was strong bootstrap support for separation of haplogroup IIa from IIb as well as haplogroup IIa and IIb from I (Fig 3). There was no bootstrap support for subgroupings of Ia and Ib within haplogroup I.

### SSR diversity

The resolving power of the 12-plex SSR assay proved appropriate for analysis of this global collection of isolates (S1 and S2 Figs). Amongst the 103 samples, 90 multi-locus genotypes (MLGs) were discriminated with a range of three (five loci) to 22 (G11) SSR alleles per locus (Table 2, S2 Fig). The three loci with the most alleles (G11, D13, and SSR4) had the most repeats (TC_60_, CT_67_, AT_20_ maximum repeat length, respectively) and the highest Simpsons Diversity index (Table 2). Null alleles were recorded in 9 isolates at the D13 locus and in one isolate at the G11 locus. In each case, null alleles were confirmed in repeat runs of the same isolate and, if relevant, in many other isolates of the same clonal lineage (data not shown). Three alleles were recorded at one or more loci in 41 of the 103 isolates and these were defined as triploid within *Polysat* [57]. A broadly similar percentage of isolates sampled in Europe (33%) and North America (42%) were defined as triploid in this way. Amongst the limited samples of nine isolates of the ‘Other’ and South American samples 11 and 89% were triploid, respectively.

**Table 2:** Summary data for each SSR locus.

Locus	Allele number	Simpson Index	Evenness
D13	16	0.81	0.62
G11	22	0.83	0.55
Pi02	5	0.60	0.76
Pi04	4	0.58	0.83
Pi4B	9	0.65	0.71
Pi63	3	0.51	0.75
Pi70	3	0.23	0.50
SSR11	3	0.62	0.92
SSR2	3	0.44	0.70
SSR4	13	0.84	0.75
SSR6	10	0.47	0.50
SSR8	3	0.59	0.87

An examination of allele frequency in relation to isolate recovery location (S2 Fig) indicated many alleles that were shared across populations of *P*. *infestans* whereas others predominated in certain geographical regions. Allele 134 at locus G11, for example, was specific to isolates sampled in North America whereas allele 162 at the same locus predominated in European isolates (S1 Fig). At locus D13, alleles from 108 to 112 bp in size were observed almost exclusively in isolates from North America. Some alleles were discrete to particular clonal lineages; for example, the 189 allele at locus Pi70 and the 177 allele at SSR2 were, with one or two exceptions, exclusive to the US-1 lineage (S3 Table).

The *poppr* summary statistics of the 42 samples from each of the North America and Europe sampling locations (Table 3) indicated that both were similar in diversity (Shannon-Wiener [60] and Stoddart and Taylor’s MLG indices [61]), Nei’s expected heterozygosity [62] and the E.5 measure of evenness [63], [64], [65].

**Table 3:** Summary statistics for each sampling location.

Loc^1^	N^2^	MLG^3^	H^4^	G^5^	Hexp^6^	E.5^7^
EU	42	39	3.64	36.75	0.997	0.965
NA	42	37	3.55	31.5	0.992	0.904
Other	9	7	1.89	6.23	0.944	0.932
SA	10	10	2.3	10	1	1
Total	103	90	4.45	79.77	0.997	0.931

^1^Loc–Location; EU = Europe, NA = North America, SA = South America

^2^N - Number of individuals observed.

^3^MLG—Number of multilocus genotypes (MLG) observed.

^4^H - Shannon-Wiener Index of MLG diversity

^5^G - Stoddart and Taylor’s Index of MLG diversity.

^6^Hexp—Nei’s expected heterozygosity (denoted elsewhere as D).

^7^E.5 - Evenness, E5.

**Fig 4:**
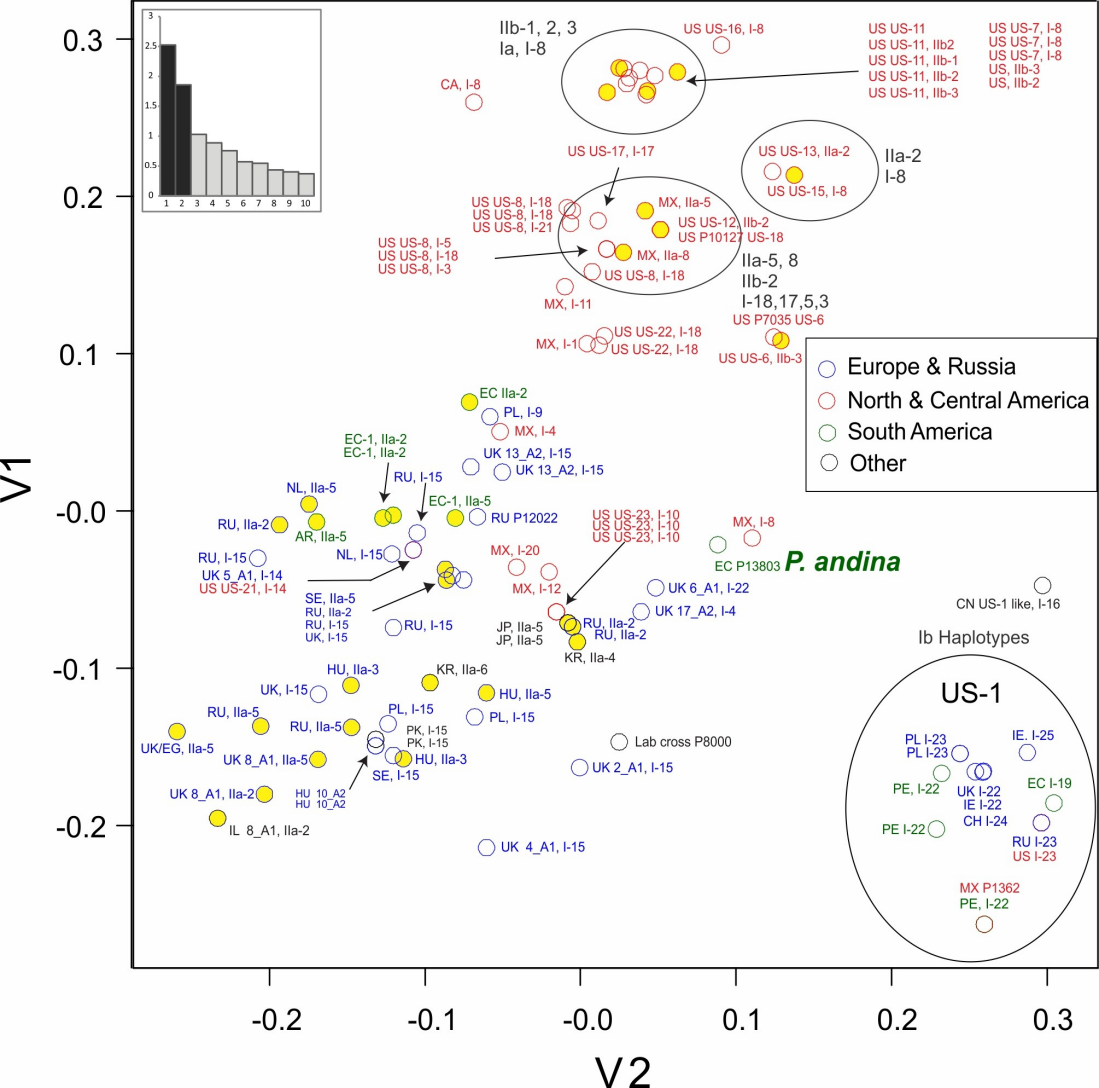
PCA of SSR data for *Phytophthora infestans* showing relationship of geographic location of recovery, clonal population (where known) and mitochondrial haplotype. Yellow circles represent haplogroup II mitochondrial types. The eigenvalue score plot indicates the variance accounted for the first ten principal components.

SSR diversity visualized by PCA analysis subdivided the isolates into 3 broad groupings (Fig 4); firstly, 14 isolates categorized as US-1, secondly a cluster comprising 32 isolates sampled in North America and thirdly a more diverse assemblage of 57 isolates collected in Europe (35), North America (8), South America (5) and the category ‘Other’ (7) (including Japan, Israel, Pakistan, South Korea and a laboratory strain). The single isolate of *P*. *andina* was placed at the margins of this latter group of 57 isolates. Greater genetic diversity was apparent amongst US-1 isolates than, for example, the cluster of isolates defined as US-11 and US-7, the two overlapping points representing isolates of US-6 or the two isolates of lineage 13_A2. The eigenvalue scree plot demonstrates that the first two principal co-ordinates account for a relatively large portion of the variance within the dataset.

**Fig 5:**
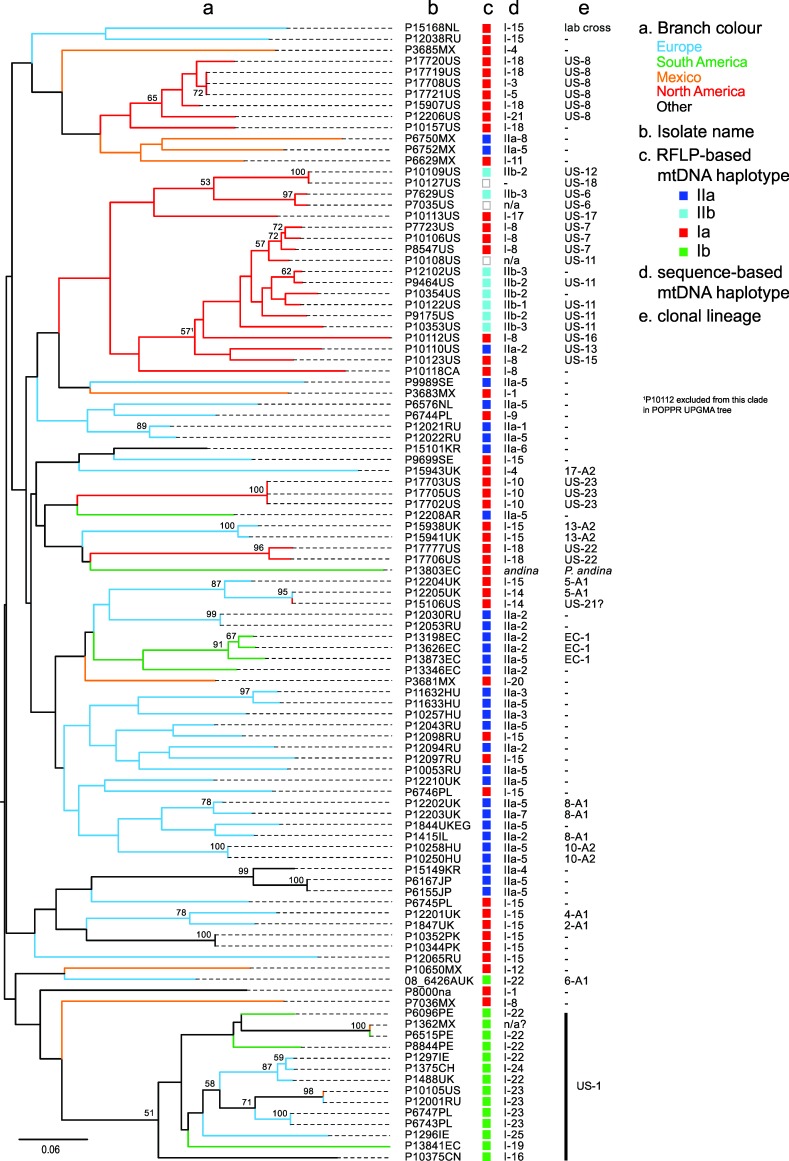
Bruvo distance NJ tree of *P*. *infestans* isolates based on 12-plex SSR data indicating sample location (branch colour), mtDNA haplotype (based on RFLP and sequencing) and the clonal lineage (where known). Bootstap values >50 are shown at nodes.

**Fig 6:**
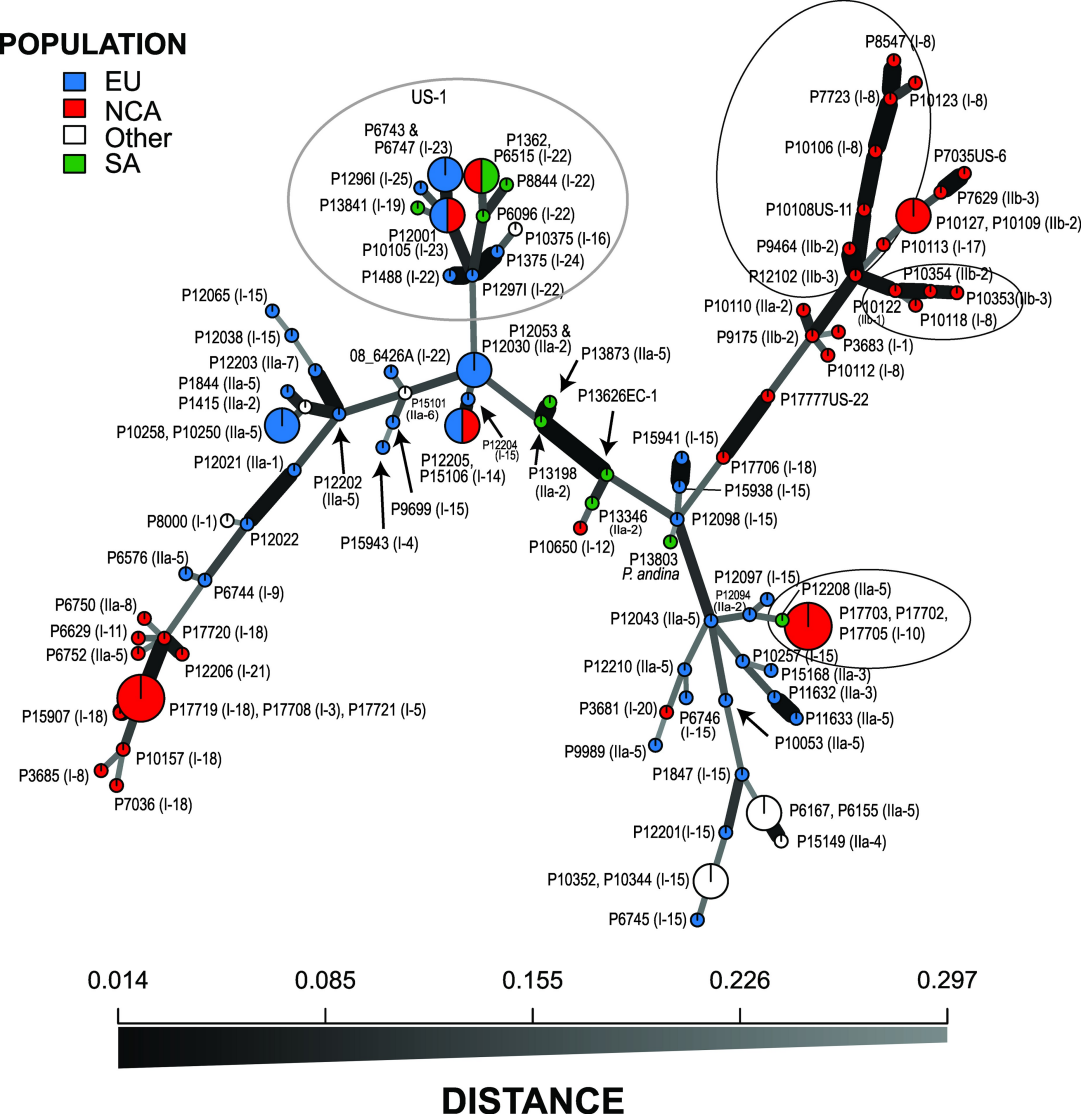
Minimum spanning network of SSR data for *Phytophthora infestans* showing isolate accession number, location of recovery and mitochondrial haplotype indicated in the brackets. Circled areas represent the branch of US-1 isolates or portions of the analysis where closely affiliated isolates based on SSR analysis have different mitochondrial haplotypes.

The neighbor-joining tree generated from Bruvo distance data (Fig 5) comprised many relatively long branches with nodes towards the root of the tree. Bootstrap support of greater than 50% was primarily for nodes at the terminal branches of groups of isolates of a single clonal lineage. Exceptions were the 51% support for a more basal node to the cluster encompassing all US-1 isolates and one of 57% that included isolates of US-7, US-11, US-13 and US-15. These two clusters are also clearly seen in the PCA analysis (Fig 4). Some relationships between geographic sample location and SSR Bruvo distance clustering were apparent (Fig 5). For example, NA clusters with representatives of the same clone forming well-supported terminal branches (e.g. US-8) and a loose association between many US and Mexican isolates. Other isolates from the US, however, group amongst isolates from EU, SA and ‘other’ regions. Isolates sampled in SA group either within the US-1 clade or form a discrete well-supported clade of three EC-1 isolates. The isolates sampled in Europe within this study are found across much of the tree, interspersed with isolates of other populations. These relationships are also observed in the network analysis of the data (Fig 6).

Within-clone diversity was examined in the 12 cases where more than one isolate of a known clonal lineage was represented (8 US-8, 2 US-6, 3 US-7, 5 US-11, 3 US-23, 2 EU_13_A2, 2 US-22, 3 EU_5_A1(&US-21), 3 EC-1, 3 EU_8_A1, 2 EU_10_A2, 14 US-1). In some cases, the SSR profiles were identical yielding a Bruvo distance of 0 but minor changes in one or more loci were also evident and within-clone Bruvo distances ranged from 0.196 in US-1, 0.054 in US-8, 0.095 in US-11, 0.090 in EU_8_A1 to 0.046 in EC-1. Over all the pairwise isolate comparisons the average distance within a clone was 0.148 compared to 0.433 between clones.

### Complementary analysis of mitochondrial haplotype and nuclear genotype

A combined view of both the nuclear SSR and cytoplasmic mtDNA results yielded novel insights into this global isolate collection (Figs 4–6). The PCA plots (Fig 4) and SSR-based tree (Fig 5), for example, show haplogroup IIb (types IIb-1 to IIb-3) had a limited distribution and contributed to only the clonal lineages US-11, US-6 and US-12. In contrast, isolates with haplogroup I or IIa were found in many SSR genotypes from multiple sample locations. Haplogroup IIa contributed to known lineages such as EC-1 and EU_8_A1 plus a single US lineage (US-13) but also to many isolates from across the SSR-based Bruvo distance tree from Europe, Mexico, Argentina and Asia (Fig 5). Haplogroup I was the most prevalent and diverse of the groups and a substructure between haplotype and SSR genotype was apparent in some cases. Of the 59 haplogroup I isolates, 18 were in a very recently diverged group (I-12 to I-15, Figs 5 and 6), fourteen of which were found amongst distinct lineages defined by SSRs but only in European samples. Indeed, haplotype I-15 contributed to lineages EU_13_A2, EU_5_A1, EU_2_A1 and EU_4_A1 as well as other isolates sampled in Europe which strongly suggests that isolates with this haplotype are prevalent in Europe and form the oogonial parent of many contemporary lineages. The PCR-RFLP type Ib (corresponding to haplotypes I-16, I-19 and I-22 to I-25) were, except for one isolate, all grouped in a single cluster of US-1 isolates (Figs 5 and 6). The exception was a European isolate of lineage EU_6_A1 that was not a US-1 isolate (Fig 6). Within the US-1 SSR clade most isolates had I-22 to I-25 mitochondrial haplotypes that formed a terminal cluster in the mtDNA network (Fig 2) but the two Ib isolates with haplotypes that joined the tree at nodes basal to this terminal cluster also had SSR variation that placed them at basal nodes of the US-1 clade (Fig 5).

In addition to the within US-1 haplotype diversity, a degree of mtDNA haplotype diversity was observed within other older clonal lineages such as EU_8_A1 (haplotypes IIa-5,7 and 2), US-8 (haplotypes I-3,5,18 and 21), US-11 (IIb-1, 2 and 3), 5-A1 (I-14 and 15), EC-1 (IIa-2 and 5) and 8-A1 (IIa-2, 5 and 7)(Fig 5, S1 Table). The expanded mitochondrial haplotype classification also helped differentiate isolates with similar SSR genotypes from the same geographic region. For examples, two isolates from Hungary (P11632 and P11633) had similar genotypes but differed in mitochondrial haplotype (IIa-3 and 5) (Figs 5 and 6); likewise for two isolates from Russia (P12021, P12022 with IIa-1 and -5, respectively). Additional grouping of isolates with a similar SSR genotype and different mitochondrial haplotype can be seen in Fig 4 (note circled areas with mixtures of type I and II mitochondrial haplotypes).

## Discussion

The objectives of this study were to develop and test a new mtDNA haplotyping system for use, in combination with an existing 12-plex SSR assay, for the discrimination and genetic analysis of a diverse assemblage of *P*. *infestans* isolates that included many dominant clonal lineages on a global scale. The mtDNA sequencing approach resolved 37 *P*. *infestans* haplotypes, a marked increase in resolution from the four haplotypes defined using the prior PCR-RFLP approach [37]. The 12-plex SSR assay discriminated 90 MLGs amongst the 103 isolates and could clearly resolve differences between clonal lineages and identify fine-scale variation within clonal lineages. The nuclear SSR and new mitochondrial markers are complementary and, when applied to appropriately sampled populations, will yield novel insights on evolutionary questions relating to the diversity, phylogeography and the origins and spread of lineages of *P*. *infestans*. Our work also proposes a new mtDNA haplogroup and haplotype terminology for the 37 haplotypes defined in this study.

The 37 multi-locus mitochondrial haplotypes for *P*. *infestans* described here can be broadly divided into three groups. Haplotype classifications of Ia and Ib were collapsed in the current analysis due to the lack of clear separation when using these five loci in network (Fig 2) and phylogenetic (Fig 3) analysis, however, the continued separation of IIa from IIb was supported by bootstrap values in these analyses. The combined haplogroup I data yielded a total of 25 multilocus haplotypes compared to eight IIa and four IIb multilocus haplotypes. Differences among isolates were due to SNPs, indels and different numbers of a 36 bp repeated sequence. When examining the haplotypes for each locus individually, locus 2 (*rns-cox2*) was the most polymorphic (total of 18 haplotypes) followed by locus 1 (*rpl5-rns*) with 8 haplotypes; locus 4 (*nad3-nad5*) was the least polymorphic with all isolates classified within two haplotypes except single examples of H3, H4 and H5.

The regions used for haplotype analysis in this study were selected on the basis of their high sequence divergence and ability to delineate haplotypes. They partially overlapped with mtDNA regions reported previously in the literature. To differentiate haplotype I from haplotype II, Griffith and Shaw [37] amplified the 3' end of the *cox1* gene, a spacer sequence and the *atp9* gene (the P4 region) and digested it with *Eco*RI; locus 3 in our study spanned this same region in addition to a portion of the *nad9* gene that included this informative *Eco*RI site, but did not include the second *Eco*RI site further upstream in the *cox1* gene used to differentiate haplotype Ic [66]. The P2 locus of Griffith and Shaw ([37]; 3' end of the *nad4* gene and 5' end of *atp1*) that differentiates Ib and IIa from the other haplotypes was not amplified in this current study. Gavino and Fry [39] described two additional Ib haplotypes in isolates from the Philippines initially described by Koh et al. [38] that had 0.4 and 0.8 kb insertions between the *rpl5* and *cox1* genes; much of both these regions were sequenced in our current study (locus 1 and 2) but these additional haplotypes were not observed with our isolates (none were from the Philippines). Wattier et al. [40] reported additional sequence polymorphisms among *P*. *infestans* isolates in *orf32* to *cox1*, *cox1* to *atp9* (same as P4 locus of [37]), *atp9* to *nad9* and *cob* to *atp1*; our locus 3 (*cox1* to *nad9*) spanned two of these regions and the SNPs they had reported were also observed in the isolates we examined. Yang et al. [41] observed differing numbers of the 36 bp repeat between *orf79* and *cox2* and proposed changing haplotype naming to include an "R" with a subscript denoting the number of times the repeat was present (1 to 3 times). This convention was not followed in our current naming system as it does not take into account SNPs and indels in other loci that were examined. In addition, in the current study the repeats were observed to be present from 1 to 4 times. However, caution should be used when relying strictly on these 36 bp repeats for haplotype classification as the number of a similar type of repeat in *Bremia lactucae* (80 bp, but in a similar location in the genome) varied among single oospore progeny isolates that inherited the same maternal mitochondrial genome (F. Martin and R. Michelmore, unpublished) suggesting such repeats may be highly mutable in *B*. *lactucae* and therefore not informative for phylogeographic analysis. Additional analysis of this locus in *P*. *infestans* is needed to evaluate the stability of this repeat over time.

Additional sequence polymorphisms in the P2 and P4 region were recently reported for a group of isolates recovered from potato in Ethiopia [42]. While the P2 locus was not sequenced in the current study, a portion of the P4 was (locus 2 in this study) and the polymorphism observed in the Ethiopian isolates from base 9964 in the *cox1* gene through the spacer were not observed in any of the *P*. *infestans* isolates or the *P*. *andina* isolate (P13803) included in this study, but were present in the type culture of *P*. *andina* (P13365; Y. Zhang and M. Coffey, unpublished).

The mitochondrial haplotypes for historical isolates recovered from herbarium specimens were classified as I-6 and I-7, closely affiliated with I-8 and I-9 in the network analysis and on the same branch as *P*. *andina* (Fig 2). Interestingly, isolate P7036 (I-8) had grouped with HERB-1 isolates in the analysis of mitochondrial genomes assembled from infected herbarium samples reported by Martin et al. [5, 6]; *P*. *infestans* isolate P13346 was also included in the HERB-1 clade but this is in contrast of the findings of this current study (P13346 was classified as haplogroup IIa-2) and another by Blair et al. [45]. The sequence differences between the HERB-1 strains and I-8 and I-9 are the number of copies of the 36 bp repeat in locus 2 (I-6 and I-7 have an equal number of copies but I-8 has one less and I-9 two less) as well as a one or two base indel in locus 1 (I-6 single base, I-9 two base) and two indels in locus 4 (5 bp in I-6, 13 bp in I-7). To get a more accurate determination of the relationship of I-8 and I-9 mitochondrial haplotypes with HERB-1 further comparisons among whole mitochondrial genome assemblies are needed.

It is recognised that *P*. *andina* is a hybrid between *P*. *infestans* and another, yet to be discovered, parental isolate [46]. Others have reported isolates of *P*. *andina* with either Ia or Ic mtDNA haplotypes [67]. The *P*. *andina* isolate examined in this study inherited its maternal mtDNA from a *P*. *infestans* parent with a haplotype shown to group with HERB-1 [6]. The *P*. *andina* Ic lineage was found to be ancestral to the Ia haplotype lineage [68].

The evolutionary history of the mtDNA haplotype previously called Ib is examined in some detail and here defined as haplogroup I types I-16, I-19 and I-22 to I-25. It is almost exclusively associated with nuclear markers characteristic of the US-1 lineage. Given the widespread distribution and prevalence of the A1 mating type US-1 lineage, the virtual absence of these haplotypes in other lineages is surprising. It suggests either a lack of opportunity or strong barriers to successful mating (pre-zygotic or post-zygotic respectively) with dominant A2 lineages that have co-infected plants alongside US-1 populations. US-1 is triploid [69] which may have acted as such a barrier to mating; it is known that progeny from crosses with triploid *P*. *infestans* isolates are less fit [70]. Host isolation on tomato or other crops is a possible factor in addition to genetic incompatibility linked to the triploid genome of US-1 which may result in progeny with reduced fitness ([70]). Kildea et al., [71] reported the presence of mtDNA type Ib in the European 6_A1 lineage first reported in 2002 [18]. In this study, we confirmed this finding and defined the haplotype as I-22, one that is typical of many US-1 haplotypes (Fig 5). Type Ib was also reported within the *P*. *infestans* population of the Netherlands [19] and it will be interesting to examine this reappearance in more detail. Isolates defined as Ib using the RFLP method (single *Msp1* restriction site in P2 region differentiating haplotype Ia from Ib) were resolved into six haplotypes using the five loci in our study but exhibited homoplasy in network analysis and could not be clearly differentiated from Ia haplotypes (Fig 2). Whole mitogenome phylogenies also support a major split between type I and type II mtDNA haplotypes, as well as separation of PCR-RFLP Ia and Ib haplotypes, and discriminate at least five clades of type I mtDNA [5, 6] that broadly correspond to the clusters seen in this study (Fig 2). One type I clade comprises 10 samples defined as US-1/Ib [nb P6096 is US-1 despite not being shown as so in [6]] and, in contrast to this study, appears distinct from other type I clades. This is likely due to the higher resolution of the almost complete mitogenome analysis compared to the five loci used in the current study. A number of contemporary and historical isolates of *P*. *infestans* have been sequenced and their mitochondrial genomes assembled [4–6, 12] but unfortunately complete assemblies are not publicly available for all isolates. Having these complete assemblies available would provide a more comprehensive approach for future optimization of mitochondrial haplotype diversity and classification.

To limit the amount of sequencing needed for classification we established an effective minimum set that defines most haplotypes. Sequencing locus 2 and 5 (*rns—cox2* and *nad6—nad4L*, respectively) will differentiate 28 haplotype groupings, within these groups there will be some that will require additional sequencing to differentiate further haplotypes (S1 Table). For example, IIa-1 and IIa-2 group together using locus 2 and 5 data (both have haplotype 3), but sequencing locus 1 will differentiate these haplotypes (this locus will also differentiate I-6 and I-7). Likewise, haplotype IIa-4 and IIa-5 group together using locus 2 and 5 data (haplotypes 5 and 3, respectively) but sequencing locus 4 will differentiate these haplotypes and sequencing locus 3 will differentiate the remaining grouped haplotypes (IIa-7 and IIa-8; I-3, I-5, I-18 and I-21; I-8 and I-22). If new haplotypes are found, it will be important to sequence all the same 5 loci as used in this study to make sure the haplotype classification is correct (a new haplotype class that is identified based on just locus 2 and 5 may actually represent multiple haplotypes due to differences in the other loci).

The 12 SSR loci used in this study resolved 90 MLGs amongst 103 isolates of diverse global origin and demonstrated the power of the method for discriminating amongst isolates and populations of *P*. *infestans* (Figs 4–6, S1 and S2). A list of allele size calls for these SSR loci and reference profiles of key clonal lineages is provided (S3 Table). This data will benefit comparisons between research teams and is consistent with that used in the international database of the EuroBlight consortium (www.euroblight.net). The 12 loci mutate at different rates (Table 2) providing ‘core’ loci that discriminate between lineages and more variable ones (e.g. G11, D13 and SSR4) that can resolve sub-clonal variants that are useful for tracking inoculum spread. The presence of triple bands in some SSR loci was consistent with mixed ploidy amongst the population. Flow cytometry [70], [72] and SNP calling after genome sequencing [4], [69] provides supporting evidence for the presence of triploid isolates.

The set of isolates in this study included several examples of two or more genetically identical (or almost identical) isolates representing clones plus many genetically distinct sexual recombinants. This pattern of mixed ploidy and the presence of clones complicates population genetic analysis [22] and is thus dependant on the genetic distance method of Bruvo implemented in *Polysat* [55], [57] and now also *poppr* [56], [73]. The 103 isolates for the current study were selected to maximise diversity within and between geographically divergent populations and the SSR diversity does not therefore represent fully the detailed population structure within each region.

The high mutation rate of these SSR loci are particularly useful for resolving relationships between closely related clones and within clonal lineages. This is apparent in the SSR diversity amongst the multiple isolates of the US-1 lineage that have been accumulating mutations for more than half a century [4] which is greater than amongst samples of US-8 which emerged and spread in the 1990s [74] or 13_A2 and US-23 clones that were first recorded in 2004 [18] and 2009 [17], respectively. Similar patterns of SSR diversity were observed in a clonal lineage of *P*. *ramorum* [75]. Within the US populations, the SSR data supports suggestions from other studies that US-7 and US-11 are related [76]. The SSRs also clearly discriminate other US and European lineages (Fig 4).

Isolates from South America, others from Asia and the *P*. *andina* isolate group more closely to isolates sampled in Europe than to those from the US or Mexico which may reflect a broad subdivision in global populations that is apparent in the allele frequency plots (S2 Fig). However, the predictive power of SSRs at accurately reflecting relatedness decreases over evolutionary time due to the unpredictability of the rate and/or direction (expansion or contraction) of stepwise SSR mutations [77], [78]. This is consistent with the weakly supported, deeper nodes in the phylogenetic tree (Fig 5). Nonetheless, broad trends were observed with most US and MX isolates forming clusters distinct from those sampled in other areas. This is consistent with a Mexican origin of recent US lineages [1], [79], [8]. A comprehensive study of historic and contemporary samples of *P*. *infestans* from Europe, US and across all regions of Mexico is required to fully understand the spatial and temporal details of the migration from Mexico to Europe.

The maternally inherited mtDNA and nuclear SSR data are complementary and illustrate the value of the combined approaches, providing insights into the phylogeography of global populations and the parentage and origin of some important pathogen clones. In agreement with other studies [39], our sample includes relatively few isolates with IIb haplotypes as maternal donors. The IIb haplogroup has been previously reported in Mexico [2] and is thus a probable source of the IIb haplotypes 1 to 4 in the US-6, US-11 and US-12 lineages. Isolates of haplogroup IIa were sampled more frequently and were the maternal parent of the lineages EU_8_A1 first reported from Europe [18], [19] and EC-1 sampled widely in South America. Clade IIa haplotypes were also found in other SSR genotypes sampled from Korea, Japan, Russia, Mexico and parts of Europe, supporting a prior widespread distribution of this mitochondrial lineage. Mutations within the SSR alleles and the mtDNA genome (Fig 5) discriminate sub-clonal variants amongst the three isolates of EU_8_A1, a lineage reported in 1995 [18], and can be used for tracking its evolution and spread. Haplogroup I isolates were the most prevalent in our collection; reported from the Americas, Europe, Asia and South America. These haplotypes have contributed maternal mtDNA to numerous contemporary and historical clones such as EU_13_A2, US-8, US-23 and US-1 as well as the HERB-1 mtDNA to the FAM lineage [11]. The mtDNA sequences in this haplogroup form clusters that broadly support a phylogeographic structure to the populations. Isolates in common between this and a near full mitogenome phylogenetic analysis published by Martin et al. [6] show parallels between the studies. Our types I-6 and I-7 represent the HERB-1 types and are closely related to haplotypes I-8 and I-9 inherited by the clones US-7, US-15 and US-16 plus one isolate each from Mexico and from Poland and the sample of *P*. *andina*. This is consistent with the HERB-1 mt haplotype clade in the Martin et al. study and both confirms and adds to the data confirming the persistence of this mtDNA lineage in contemporary populations [5]. Another cluster, I-16 to I-21, is also found predominantly in US lineages such as US-8 and US-22 and is consistent with a clade of 3 isolates termed AGG in the study of Martin et al. [6]. Conversely, isolates with the group of mtDNA variants from I-10 to I-15 must have been the maternal parents for clones sampled first from Europe including EU_13_A2, EU_2_A1 and EU_5_A1 plus many other isolates of predominantly European origin. This group corresponds to the lower sub-clade marked Ia in the Martin et al. analysis [6]. Lastly, the cluster of I-22 to I-25 mtDNA sequences was almost exclusively found in the US-1 clonal lineage (see previous discussion). In addition to the above associations between nuclear SSR data and mtDNA haplotype there are cases where unrelated SSR genotypes sampled from different continents share identical mtDNA haplotypes (e.g. I-15 and IIa-5; Figs 5 and 6, S1 Table). This is entirely consistent with the independent maternal inheritance of mtDNA haplotypes and their nuclear SSR alleles in mating metapopulations of *P*. *infestans* with different mixes of mtDNA haplogroups.

The analysis of the selection pressures shaping such populations of *P*. *infestans* will be advanced by studies that include both these sets of nuclear and cytoplasmic markers which provide complementary perspectives of pathogen evolution. The multilocus mitochondrial haplotype classification system provides a greater level of resolution for differentiating isolates than the traditional PCR-RFLP technique, with the Ia PCR-RFLP haplotype expanded to 17 haplotypes, Ib expanded to 6, IIa expanded to 8 and IIb expanded to 4 for the 108 isolates included in this study. Given the level of sequence divergence observed thus far, it is likely that examination of additional isolates will increase the number of mitochondrial haplotypes and further support use of these markers for population studies.

## Supporting information

S1 Table Cultures used in this investigation, mating type, historical mitochondrial haplotype, genotype and mitochondrial haplogroup. (XLSX)

S2 Table GenBank accession numbers for the five mitochondrial loci used for haplogroup analysis. (DOC)

S3 Table SSR data for the 12 loci used in this study. (XLSX)

S1 Fig Genotype accumulation curve for 103 isolates of *P. infestans* generated in *poppr*. The vertical axis shows the number of multilocus genotypes up to the maximum of 90 defined in the dataset and the horizontal axis the number of loci sampled without replacement up to *n–* 1 loci.
(DOCX)

S2 Fig Allele frequency figure. (DOCX)

S3 Fig PCA of SSR data for *Phytophthora infestans* showing relationship of geographic location of recovery, Isolate number and clonal population (where known). (DOCX)

S1 File Concatenated DNA sequence alignment for loci 1 (base 1–3473), 3 (base 3474–4275), 4 (base 4276–4655), 5 (base 4656–5110), and 2 (base 5111–6087). (MSF)
