# Supplementary material for: Insights into evolving global populations of Phytophthora infestans via new complementary mtDNA haplotype markers and nuclear SSRs
Source: PLoS One. 2019 Jan 2;14(1):e0208606. doi: 10.1371/journal.pone.0208606 (PMC6314598; doi:10.1371/journal.pone.0208606)
Supplement: S2 Table — (DOC) [file pone.0208606.s002.doc]

Table2. The accession numbers of isolates used in this study.

|  | P# | Dilution # | Accession number | | | | |
| --- | --- | --- | --- | --- | --- | --- | --- |
|  |  |  | Region 1 | Region2 | Region3 | Region4 | Region5 |
| 1 | P10650 | A847 | HM627763 | HM749083 | HM749188 | HM229199 | HM138387 |
| 2 | P10110 | A1131 | HM627781 | HM749119 | HM749178 | HM229189 | HM138377 |
| 3 | P12038 | A1280 | HM627764 | HM749093 | HM749193 | HM229204 | HM138392 |
| 4 | P12053 | A1322 | HM627782 | HM749094 | HM749195 | HM229206 | HM138394 |
| 5 | P12021 | A1331 | HM627783 | HM749092 | HM749191 | HM229202 | HM138390 |
| 6 | P12017 | A1402 | HM627784 | HM749087 | HM749190 | HM229201 | HM138389 |
| 7 | P12043 | A1407 | HM627785 | HM749109 | HM749194 | HM229205 | HM138393 |
| 8 | P7629 | A1459 | HM627786 | HM749115 | HM749220 | HM229231 | HM138419 |
| 9 | P12102 | A1461 | HM627787 | HM749095 | HM749196 | HM229207 | HM138395 |
| 10 | P6576 | A1949 | HM627788 | HM749100 | HM749214 | HM229225 | HM138413 |
| 11 | P6746 | A1984 | HM627771 | HM749102 | HM749216 | HM229227 | HM138415 |
| 12 | P3685 | B58 | HM627767 | HM749086 | HM749210 | HM229221 | HM138409 |
| 13 | P6752 | B59 | HM627798 | HM749104 | HM749218 | HM229229 | HM138417 |
| 14 | P6096 | B60 | HM627768 | HM749121 | HM749211 | HM229222 | HM138410 |
| 15 | P10113 | B78 | HM627759 | HM749114 | HM749179 | HM229190 | HM138378 |
| 16 | P9464 | B87 | HM627804 | HM749107 | HM749223 | HM229234 | HM138422 |
| 17 | P10127 | B89 | HM627777 | HM749088 | HM749183 | HM229194 | HM138382 |
| 18 | P8844 | B99 | HM627774 | HM749106 | HM749222 | HM229233 | HM138421 |
| 19 | P9989 | B104 | HM627789 | HM749108 | HM749224 | HM229235 | HM138423 |
| 20 | P10122 | B122 | HM627790 | HM749120 | HM749181 | HM229192 | HM138380 |
| 21 | P10118 | B139 | HM627760 | HM749118 | HM749180 | HM229191 | HM138379 |
| 22 | P10257 | B191 | HM627791 | HM749117 | HM749186 | HM229197 | HM138385 |
| 23 | P10250 | B218 | HM627792 | HM749113 | HM749185 | HM229196 | HM138384 |
| 24 | P10344 | B252 | HM627762 | HM749090 | HM749187 | HM229198 | HM138386 |
| 25 | P1415 | B257 | HM627793 | HM749075 | HM749204 | HM229215 | HM138403 |
| 26 | P1362 | C749 | HM627778 | HM749084 | HM749200 | HM229211 | HM138399 |
| 27 | P6750 | B374 | HM627794 | HM749103 | HM749217 | HM229228 | HM138416 |
| 28 | P10123 | B376 | HM627761 | HM749081 | HM749182 | HM229193 | HM138381 |
| 29 | P10157 | B384 | HM627775 | HM749082 | HM749184 | HM229195 | HM138383 |
| 30 | P6167 | B394 | HM627795 | HM749116 | HM749212 | HM229223 | HM138411 |
| 31 | P12030 | B421 | HM627796 | HM749112 | HM749192 | HM229203 | HM138391 |
| 32 | P1844 | B428 | HM627797 | HM749099 | HM749208 | HM229219 | HM138407 |
| 33 | P7036 | B449 | HM627772 | HM749080 | HM749219 | HM229230 | HM138418 |
| 34 | P6629 | B453 | HM627770 | HM749101 | HM749215 | HM229226 | HM138414 |
| 35 | P10053 | C196 | HM749067 | HM749074 | HM749177 | HM229188 | HM138376 |
| 36 | P3681 | B463 | HM627776 | HM749085 | HM749209 | HM229220 | HM138408 |
| 37 | P1375 | B464 | HM627765 | HM749097 | HM749201 | HM229212 | HM138400 |
| 38 | P13346 | B728 | HM627799 | HM749111 | HM749198 | HM229209 | HM138397 |
| 39 | P13873 | B789 | HM627801 | HM749098 | HM749203 | HM229214 | HM138402 |
| 40 | P13626 | B790 | HM627802 | HM749096 | HM749199 | HM229210 | HM138398 |
| 41 | P13198 | B822 | HM627803 | HM749089 | HM749197 | HM229208 | HM138396 |
| 42 | P11633 | B927 | HM627805 | HM749091 | HM749189 | HM229200 | HM138388 |
| 43 | P7722 | B952 | HM627773 | HM749105 | HM749221 | HM229232 | HM138420 |
| 44 | P13841 | B953 | HM627766 | HM749110 | HM749202 | HM229213 | HM138401 |
| 45 | P6515 | B1069 | HM627769 | HM749079 | HM749213 | HM229224 | HM138412 |
| 46 | P15101 | D195 | HM627806 | HM749076 | HM749205 | HM229216 | HM138404 |
| 47 | P15168 | D1187 | HM627779 | HM749077 | HM749206 | HM229217 | HM138405 |
| 48 | P15941 | D1328 | HM627780 | HM749078 | HM749207 | HM229218 | HM138406 |
| 49 | P6743 | A968 | HM627813 | HM749130 | HM749233 | HM229244 | HM138432 |
| 50 | P10353 | A1094 | HM627835 | HM749122 | HM749225 | HM229236 | HM138424 |
| 51 | P12098 | A1104 | HM627807 | HM749123 | HM749226 | HM229237 | HM138425 |
| 52 | P10105 | A1129 | HM627808 | HM749124 | HM749227 | HM229238 | HM138426 |
| 53 | P12065 | A1268 | HM627809 | HM749125 | HM749228 | HM229239 | HM138427 |
| 54 | P12094 | A1274 | HM627836 | HM749126 | HM749229 | HM229240 | HM138428 |
| 55 | P12001 | A1318 | HM627810 | HM749127 | HM749230 | HM229241 | HM138429 |
| 56 | P12097 | A1377 | HM627811 | HM749128 | HM749231 | HM229242 | HM138430 |
| 57 | P7723 | A1586 | HM627812 | HM749129 | HM749232 | HM229243 | HM138431 |
| 58 | P6744 | B3 | HM627821 | HM749141 | HM749244 | HM229255 | HM138443 |
| 59 | P3683 | B77 | HM627829 | HM749156 | HM749259 | HM229270 | HM138456 |
| 60 | P8547 | B152 | HM627815 | HM749132 | HM749235 | HM229246 | HM138434 |
| 61 | P10258 | B206 | HM627838 | HM749137 | HM749240 | HM229251 | HM138439 |
| 62 | P10375 | B241 | HM627819 | HM749138 | HM749241 | HM229252 | HM138440 |
| 63 | P10354 | B242 | HM627839 | HM749139 | HM749242 | HM229253 | HM138441 |
| 64 | P10352 | B248 | HM627820 | HM749140 | HM749243 | HM229254 | HM138442 |
| 65 | P1296 | B300 | HM627822 | HM749142 | HM749245 | HM229256 | HM138444 |
| 66 | P1847 | B342 | HM627823 | HM749143 | HM749246 | HM229257 | HM138445 |
| 67 | P1488 | B346 | HM749066 | HM749144 | HM749247 | HM229258 | HM138446 |
| 68 | P6747 | B1677 | HM627817 | HM749134 | HM749237 | HM229248 | HM138436 |
| 69 | P1297 | B348 | HM627824 | HM749145 | HM749248 | HM229259 | HM138447 |
| 70 | P9699 | B354 | HM627825 | HM749146 | HM749249 | HM229260 | HM138448 |
| 71 | P10109 | B407 | HM627840 | HM749147 | HM749250 | HM229261 | HM138449 |
| 72 | P8000 | B438 | HM627826 | HM749148 | HM749251 | HM229262 | HM138450 |
| 73 | P10112 | B1666 | HM627815 | HM749133 | HM749236 | HM229247 | HM138435 |
| 74 | P13803 | B621 | HM627827 | HM749149 | HM749252 | HM229263 | HM138475 |
| 75 | P12202 | B636 | HM627841 | HM749150 | HM749253 | HM229264 | HM138451 |
| 76 | P12203 | B663 | HM627842 | HM749151 | HM749254 | HM229265 | HM138452 |
| 77 | P12210 | B687 | HM627843 | HM749152 | HM749255 | HM229266 | HM138453 |
| 78 | P12208 | B714 | HM627844 | HM749153 | HM749256 | HM229267 | HM138454 |
| 79 | P12205 | B727 | HM627828 | HM749154 | HM749257 | HM229268 | HM138455 |
| 80 | P12204 | B753 | HM627800 | HM749155 | HM749258 | HM229269 | HM749070 |
| 81 | P11632 | B912 | HM627845 | HM749157 | HM749260 | HM229271 | HM138457 |
| 82 | P12201 | B967 | HM627830 | HM749158 | HM749261 | HM229272 | HM138458 |
| 83 | P9175 | B997 | HM627846 | HM749159 | HM749262 | HM229273 | HM138459 |
| 84 | P12206 | B1011 | HM627814 | HM749131 | HM749234 | HM229245 | HM138433 |
| 85 | P15106 | B1761 | HM627818 | HM749135 | HM749238 | HM229249 | HM138437 |
| 86 | P15149 | B1931 | HM627837 | HM749136 | HM749239 | HM229250 | HM138438 |
| 87 | P6745 | C258 | HM627831 | HM749160 | HM749263 | HM229274 | HM138460 |
| 88 | P6155 | C261 | HM627847 | HM749161 | HM749264 | HM229275 | HM138461 |
| 89 | P15907 | D16 | HM627832 | HM749162 | HM749265 | HM229276 | HM138462 |
| 90 | P15938 | D1661 | HM627833 | HM749163 | HM749266 | HM229277 | HM138463 |
| 91 | P15943 | D674 | HM627834 | HM749164 | HM749267 | HM229278 | HM138464 |
| 92 | P17705 | D1806 | HM627848 | HM749165 | HM749268 | HM229279 | HM138465 |
| 93 | P17702 | D1807 | HM627849 | HM749166 | HM749269 | HM229280 | HM138466 |
| 94 | P17703 | D1808 | HM627850 | HM749167 | HM749270 | HM229281 | HM138467 |
| 95 | P17706 | D1809 | HM627851 | HM749168 | HM749271 | HM229282 | HM138468 |
| 96 | P17719 | D1810 | HM627852 | HM749169 | HM749272 | HM229283 | HM138469 |
| 97 | P17720 | D1811 | HM627853 | HM749170 | HM749273 | HM229284 | HM138470 |
| 98 | P17777 | D1812 | HM627854 | HM749171 | HM749274 | HM229285 | HM138471 |
| 99 | P17708 | D1797 | HM627856 | HM749172 | HM749275 | HM229287 | HM749069 |
| 100 | P17721 | D1792 | HM627857 | HM749173 | HM749276 | HM229286 | HM749068 |
| 101 | P10106 | B140 | HM627855 | HM749175 | HM749278 | HM229289 | HM138473 |
