# Supplementary figures and images for: Insights into evolving global populations of Phytophthora infestans via new complementary mtDNA haplotype markers and nuclear SSRs
Source: PLoS One. 2019 Jan 2;14(1):e0208606. doi: 10.1371/journal.pone.0208606 (PMC6314598; doi:10.1371/journal.pone.0208606)

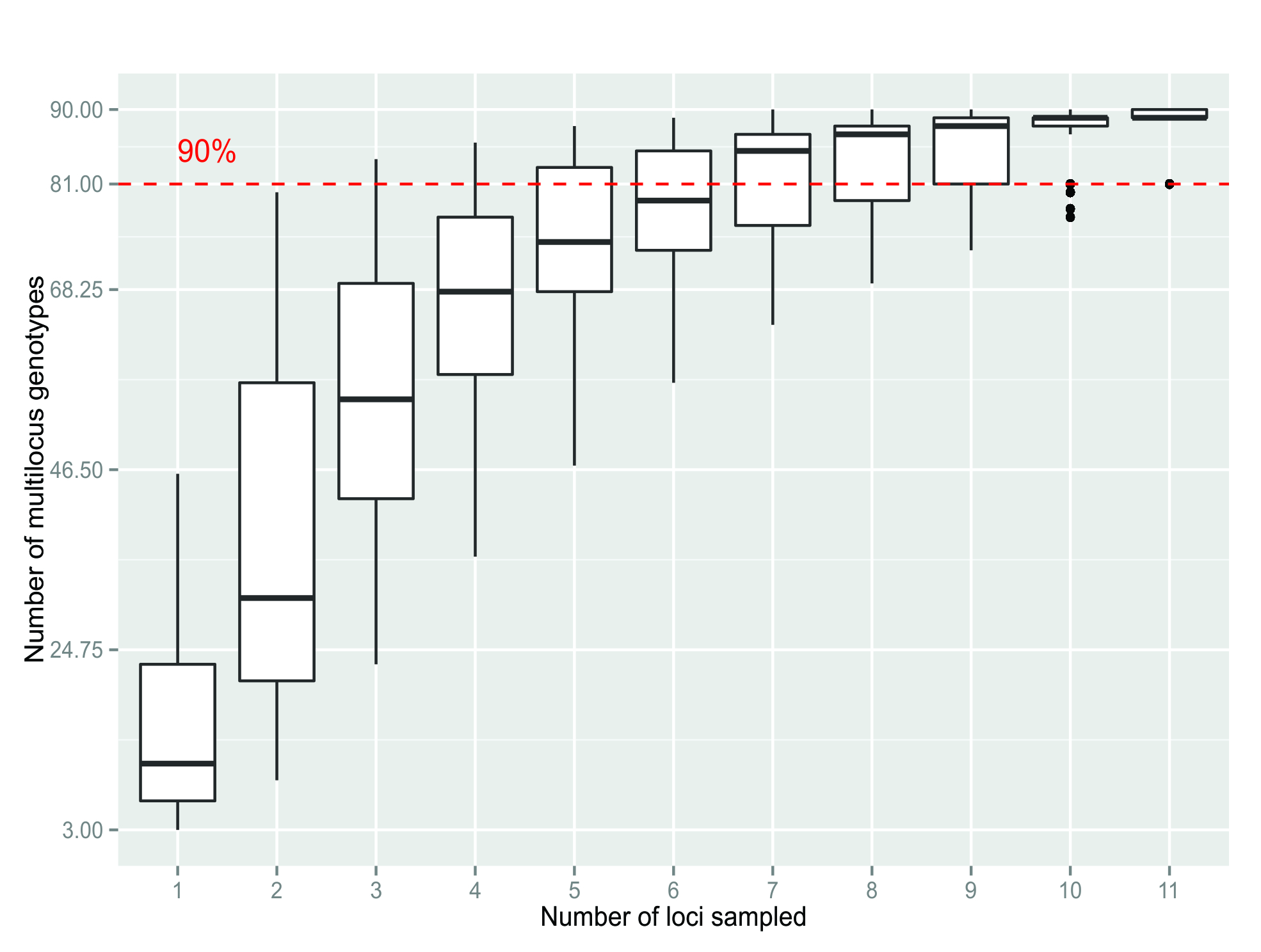
**Fig S1. Genotype accumulation curve generated using *poppr***

Supplement: S1 Fig — The vertical axis shows the number of multilocus genotypes up to the maximum of 90 defined in the dataset and the horizontal axis the number of loci sampled without replacement up to n– 1 loci. (DOCX) [file pone.0208606.s004.docx]
