# Supplementary material for: Insights into evolving global populations of Phytophthora infestans via new complementary mtDNA haplotype markers and nuclear SSRs
Source: PLoS One. 2019 Jan 2;14(1):e0208606. doi: 10.1371/journal.pone.0208606 (PMC6314598; doi:10.1371/journal.pone.0208606)
Supplement: S2 Fig — (DOCX) [file pone.0208606.s005.docx]

**.**
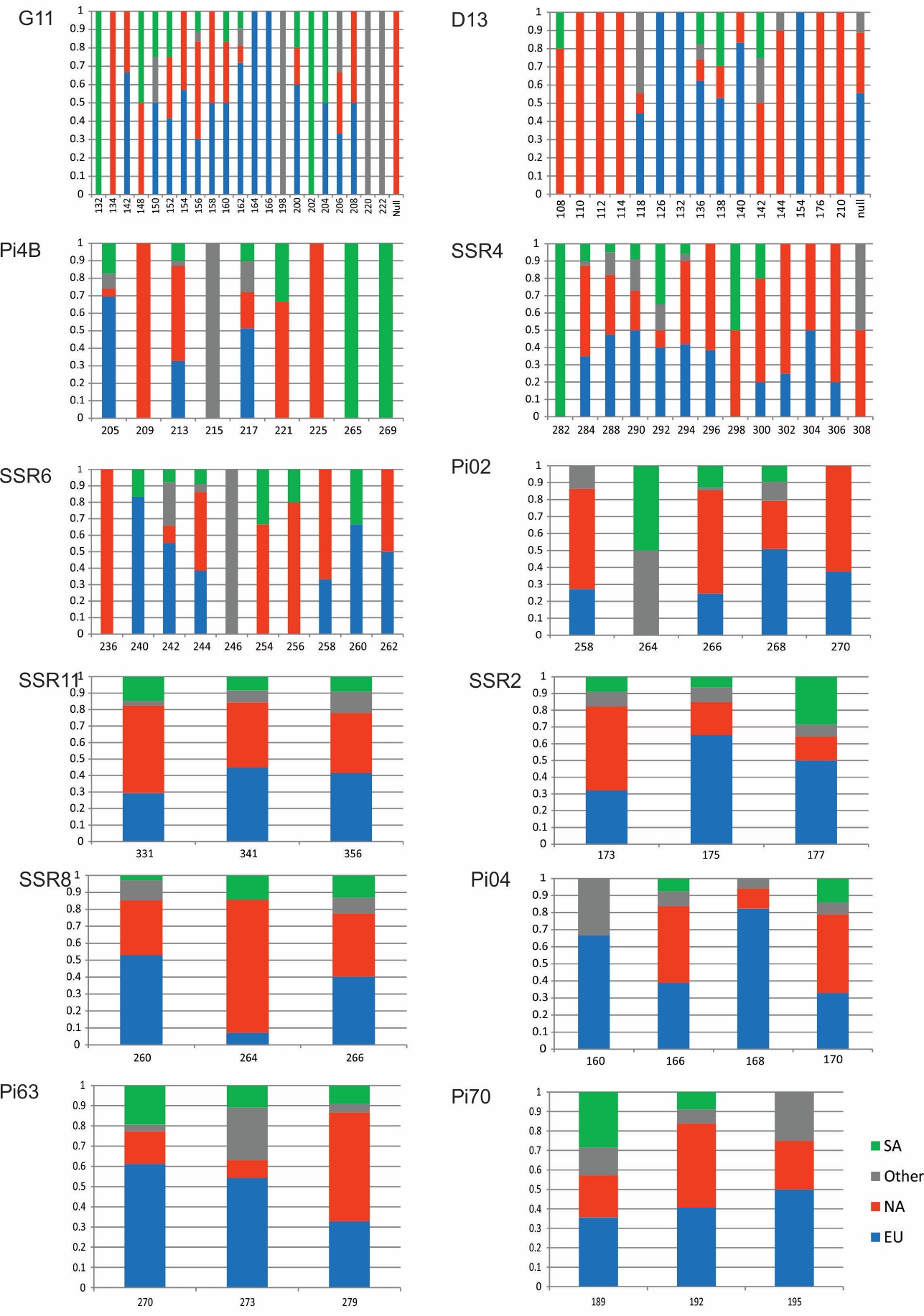


**Fig S2. Allele frequency of each of the 12 SSR loci plotted according to the geographical region the sampled isolates**
