# Supplementary material for: Insights into evolving global populations of Phytophthora infestans via new complementary mtDNA haplotype markers and nuclear SSRs
Source: PLoS One. 2019 Jan 2;14(1):e0208606. doi: 10.1371/journal.pone.0208606 (PMC6314598; doi:10.1371/journal.pone.0208606)
Supplement: S3 Fig — (DOCX) [file pone.0208606.s006.docx]

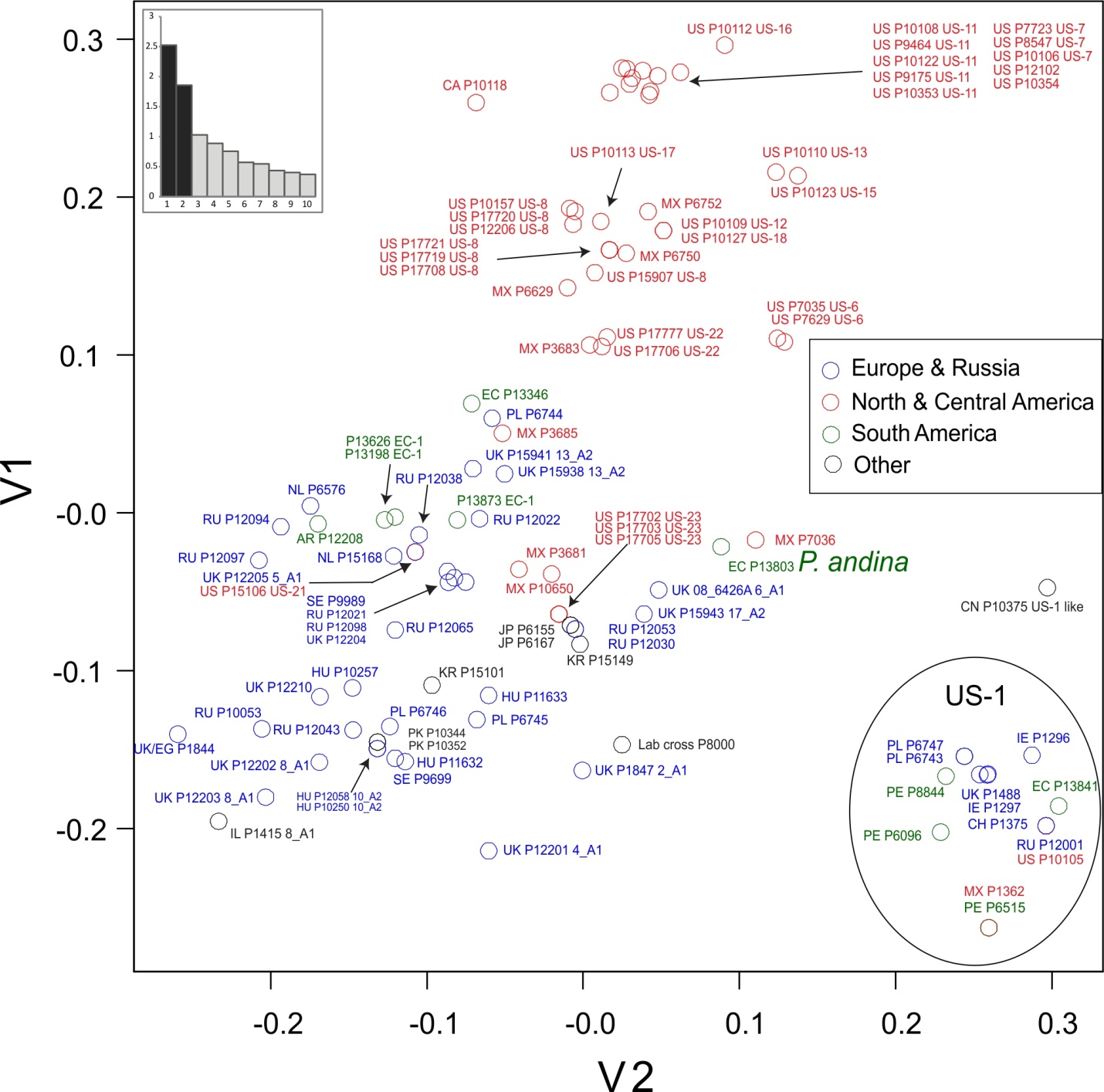


**Fig S3. PCA of SSR data for *Phytophthora infestans* showing relationship of geographic location of recovery, Isolate number and clonal population (where known).**
